# Case report: Uncovering hidden glucose patterns in medicated versus unmedicated bipolar disorder and comorbid type 1 diabetes mellitus

**DOI:** 10.3389/fendo.2024.1354749

**Published:** 2024-02-14

**Authors:** Dagmar Breznoscakova, Maria Pallayova

**Affiliations:** ^1^ Department of Social and Behavioural Medicine, Pavol Jozef Safarik University Faculty of Medicine, Kosice, Slovakia; ^2^ Center for Mental Functions, Vranov nad Toplou, Slovakia; ^3^ Department of Human Physiology, Pavol Jozef Safarik University Faculty of Medicine, Kosice, Slovakia; ^4^ 1^st^ Department of Psychiatry, University Hospital of Louis Pasteur, Kosice, Slovakia

**Keywords:** bipolar disorder, type 1 diabetes mellitus, case report, continuous glucose monitoring, quetiapine

## Abstract

**Introduction:**

Type 1 diabetes mellitus is characterized by an absolute insulin deficiency requiring the lifetime intensive insulin therapy accompanied by daily self-monitoring, self-management, ongoing education, and complex diabetes care. Regular patient-clinician shared therapeutic decisions based on age, sex, comorbidities, medications, predicted impact of meals, physical activity, stress, hormonal changes, insulin therapy, and patterns of glycemic changes are key for achieving glycemic targets. The impact of various phases of bipolar disorder and their treatment on continuous glucose levels remains unexplored and calls for future assessments.

**Case presentation:**

The present case reports a 41-year-old Caucasian female with an established diagnosis of bipolar II disorder and type 1 diabetes mellitus who discontinued long-term mood-stabilizing pharmacotherapy with quetiapine. Real-time continuous glucose monitoring performed before and 6-months following the discontinuation of quetiapine revealed hidden glucose patterns in medicated versus unmedicated bipolar disorder. Despite the known adverse metabolic effects of quetiapine, the continuous glucose monitoring captured more stable and near-normal continuous glucose values during the antipsychotic treatment compared to unmedicated stages of bipolar disorder with considerably higher glucose values and glucose variability.

**Conclusion:**

The case report highlights the importance of the ongoing psychopharmacotherapy of bipolar disorder in comorbid type 1 diabetes mellitus to reduce mood-induced reactivity, emotional urgency, and non-emotional impulsivity that may contribute to dysglycemia. If not effectively treated, the “bipolar diabetes” is likely to progress to multiple psychiatric and somatic complications. The bidirectional links between the phases of bipolar disorder and the corresponding continuous glucose patterns can help advance clinical decision-making and yield innovative1 research that can translate into efficacious clinical practice.

## Introduction

Type 1 diabetes mellitus is predominantly an autoimmune disease (accounting for around 10% of all diabetes cases) characterized by an absolute insulin deficiency requiring the lifetime intensive insulin replacement therapy accompanied by the daily self-monitoring, self-management, ongoing education, and complex diabetes care. Without proper treatment, the condition becomes life-threatening. To achieve glycemic targets, the patient-clinician shared therapeutic decisions must be made regularly based on age, sex, comorbidities, medications, predicted impact of meals, physical activity, stress, hormonal changes, type/ways/characteristics of the administered insulin, and patterns of glycemic changes detected by self-monitoring of blood glucose and/or continuous glucose monitoring.

While type 1 diabetes mellitus is often complicated by other autoimmune disorders and somatic comorbidities, less attention is paid to an unexplored impact of comorbid psychiatric disorders on glucose levels. Specifically, the impact of various phases of bipolar disorder and their treatment on dysglycemia poses a challenge, both for persons with type 1 diabetes mellitus and for their health-care providers. Bipolar disorder is a serious lifelong affective disorder characterized by periodic mood dysregulation with elevated mood states (episodes of mania or hypomania, or episodes with mixed features) with or without depressed mood states and energy levels (1). Bipolar II disorder is one of the types of bipolar disorder (2) marked by at least one hypomanic episode, at least one major depressive episode, and the absence of manic episodes.

Bipolar disorder symptoms and emotional urgency can potentially compromise diabetes control, particularly in brittle diabetes. Despite an evidence that stress hyperglycemia is associated with poor clinical outcomes (3), the extent to which the bipolar mood-induced reactivity may affect actual glucose levels and glucose variability in type 1 diabetes mellitus is still undetermined and calls for future assessments. The present case reports the baseline and follow-up results of continuous glucose monitoring in a middle-aged female with bipolar II disorder and type 1 diabetes mellitus who discontinued long-term mood-stabilizing antipsychotic treatment. Particularly unique about the present case is the ability to uncover the hidden glucose excursions in medicated vs. unmedicated bipolar disorder, which is relevant for clinical practice. Novel insights may help to clarify the links between mood fluctuations and associated glycemic fluctuations. The findings will thus inform clinical practice and further research to continue to investigate novel approaches to prevent adverse outcomes in bipolar disorder and comorbid type 1 diabetes mellitus.

## Case presentation

Ms. K. is a 41-year-old Caucasian female teacher who has been attending mental health services for almost 6 years and has an established diagnosis of bipolar II disorder. She has previously experienced several episodes of illness and undergone one previous hospitalization with severe depression. Her illness has been stable for the past five years since commencing antipsychotic treatment. For the past four years she has been on a monotherapy with quetiapine. The participant’s race, ethnicity, female sex, and female gender identity were ascertained by self-report.

Her past medical history (**Table 1**) included brittle type 1 diabetes mellitus (since 1996) without significant complications that was well-managed with an insulin pump therapy, Hashimoto thyroiditis (since 1996) with hypothyroidism (since 2014), arterial hypertension (since 1998), and antiphospholipid syndrome (since 2023). Owing to the presence of multiple autoimmune diseases and brittle type 1 diabetes, the patient has been fully assessed. An immunological examination detected negative anti-21-hydroxylase autoantibodies, negative antiparietal cell antibodies, negative antibodies against extractable nuclear antigen and positive titers of antibodies to glutamic acid decarboxylase, antithyroid peroxidase and anti-thyroglobulin in the serum. There were normal levels of vitamin B12, normal blood count results, and no symptoms of malabsorption. The results confirmed the presence of the immune-mediated diabetes mellitus with autoimmune thyroiditis (Polyglandular Autoimmune Syndrome type IIIA). The patient was single, denied recreational drug use, did not smoke, and only drank alcohol occasionally. She was highly educated. She was employed as a full-time teacher at the local university. There was a family history of type 1 diabetes mellitus, type 2 diabetes mellitus, arterial hypertension, and coronary artery disease. Family psychiatric history was positive for cyclothymic disorder, impulsive borderline personality disorder, depressive disorders, and alcohol use disorders.

**Table 1:** Past medical history and current treatment.

past medical history	year of diagnosis	current treatment
type 1 diabetes mellitus	1996	insulin pump therapy, insulin lispro
diabetic axonal polyneuropathy	2012	alpha-lipoic acid, benfotiamine
Hashimoto thyroiditis	1996	none
primary hypothyroidism	2014	levothyroxine
arterial hypertension	1998	bisoprolol
bipolar disorder	2018	quetiapine SR
antiphospholipid syndrome	2023	none

**Figure 1:**
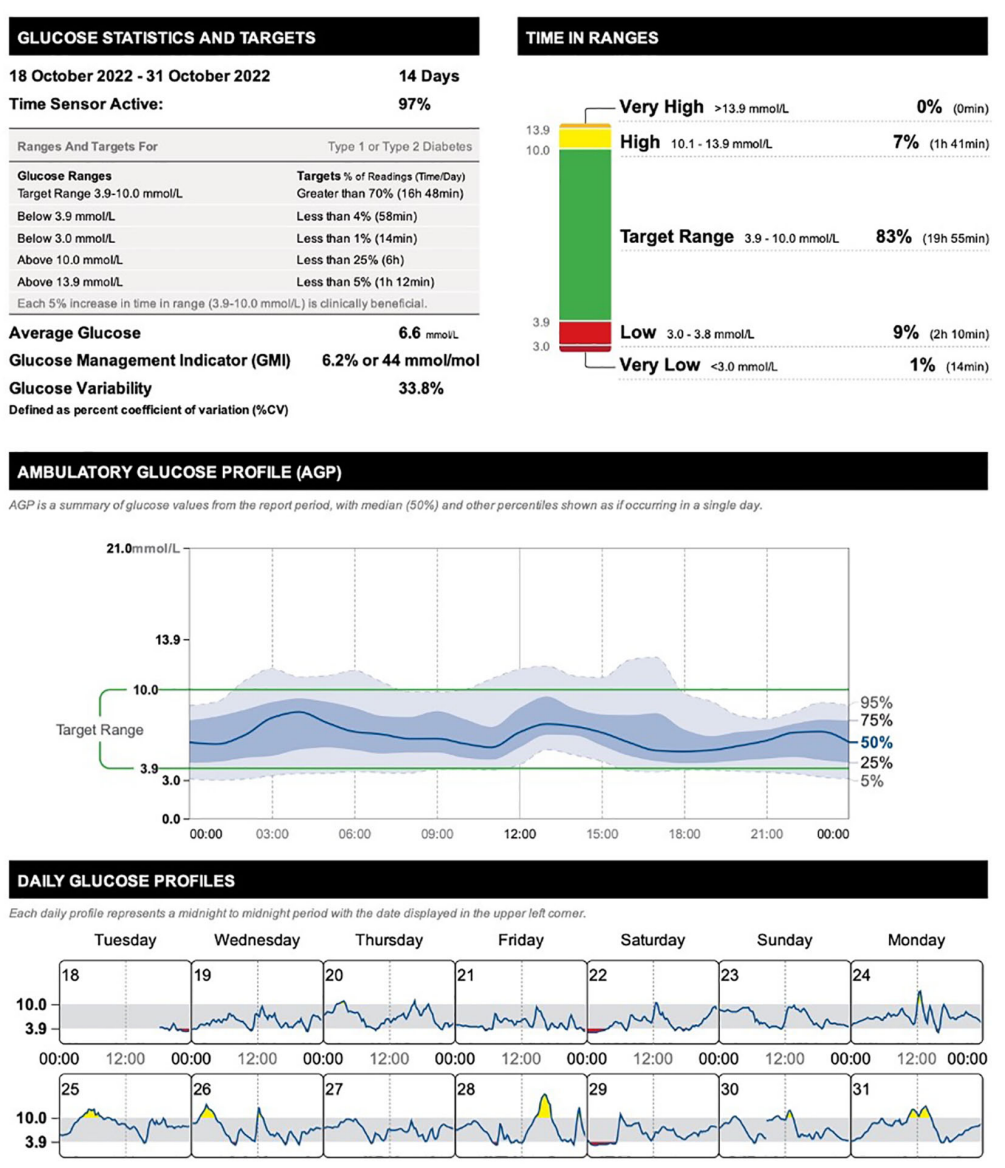
Summary of the continuous glucose monitoring results reflecting the period of medicated bipolar disorder (quetiapine SR 300 mg nightly) in a 41-year-old female with comorbid type 1 diabetes mellitus.

Since January 2019 till January 2023, the patient’s chronic medication therapy included insulin lispro 30-40 IU/day delivered via insulin pump at preset basal rates (40%) and administered in several premeal boluses (60%), levothyroxine 100-125 mcg q.d., bisoprolol 5 mg q.d., and quetiapine SR 200-400 mg q.d. (nightly) (**Table 1**). Until 2021 she also had aripiprazole 10-30 mg q.d. Her adherence to therapy was excellent. Her diabetes was well-controlled with glycated hemoglobin (HbA1c) values near normal range during the past five years. Her most recent laboratory NGSP HbA1c readings were 6.10% (IFCC HbA1c 43 mmol/mol) in June 2022 and 5.77% (40 mmol/mol) in November 2022. Because of the intensive insulin treatment, she self-monitored her blood glucose daily and regularly performed the real-time continuous glucose monitoring using FreeStyle Libre^®^ system to reveal and manage even hidden glucose excursions. The system is minimally invasive and uses a disposable factory-calibrated glucose sensor inserted into the user’s arm. A touchscreen device/reader is then used to scan and retrieve continuously monitored real-time glucose readings. **Figure 1** depicts the summary of the continuous glucose monitoring results reflecting the period of consecutive 14 days (October 18-31, 2022) of medicated bipolar disorder. The patient met important glycemic targets, including the time within target range (83%), time above 10.0 mmol/l (7%), and time above 13.9 mmol/l (0%). The average sensor glucose was 6.6 mmol/l, glucose management indicator 6.2% (44 mmol/mol), and glucose variability was 33.8% (defined as percent coefficient of variation). **Figure 1** also shows an ambulatory glucose profile, a summary of sensor glucose values from the report period, with median and other percentiles shown as if occurring in a single day. Finally, the **Figure 1** provides details on daily glucose profiles with each daily profile representing a midnight-to-midnight period. During this monitoring, the patient’s mood was stable, and she was on bimodal mood-stabilizing monotherapy with quetiapine SR 300 mg q.d. (nightly). Other prescribed drugs included levothyroxine 125 mcg q.d., bisoprolol 5 mg q.d., alpha-lipoic acid 600 mg q.d., and benfotiamine 300 mg q.d. On physical examination, her height was 170 cm, and her weight was 63 kg. The body mass index was 21.8 kg/m². Her body weight was stable over the past three years. Thyroid function tests and the baseline laboratory findings were normal.

In December 2022, her mood deteriorated with a rapid onset of severe depression requiring immediate management plan (**Table 2**). At the review, it emerges that the depressive episode was triggered by a newly diagnosed health problem the patient does not wish to be disclosed in this report. The patient is not keen to be admitted to hospital, and her friend volunteers to “keep an eye on her for a few days.” After discussion, she agrees to have her symptoms managed as an outpatient, and she agrees to gradually increase her dose of quetiapine to a therapeutic dose. Over the coming weeks, her low mood improves, and her mental state settles with quetiapine SR 800 mg nightly.

**Table 2:** A timeline of relevant patient-related data from each episode of care.

**January 2019 - January 2023**	• insulin lispro 30-40 IU/day • levothyroxine 100-125 mcg q.d. • bisoprolol 5 mg q.d. • alpha-lipoic acid 600 mg q.d. • benfotiamine 300 mg q.d. • quetiapine SR 200-400 mg q.d. (nightly) • aripiprazole 10-30 mg q.d. (until 2021)	• excellent adherence to therapy • HbA1c 6.10% (43 mmol/mol) in June 2022 • HbA1c 5.77% (40 mmol/mol) in November 2022 • regular self-monitoring and continuous glucose monitoring (CGM) with excellent results
**October 18-31, 2022**	• insulin lispro 30-40 IU/day • levothyroxine 125 mcg q.d. • bisoprolol 5 mg q.d. • alpha-lipoic acid 600 mg q.d. • benfotiamine 300 mg q.d. • quetiapine SR 300 mg q.d. (nightly)	• CGM time within target range 83%, time above 10.0 mmol/l 7%, time above 13.9 mmol/l 0% • average sensor glucose 6.6 mmol/l, glucose management indicator 6.2% (44 mmol/mol), glucose variability 33.8%
**December 2022**	• ongoing diabetes, antihypertensive and thyroid treatment • quetiapine SR dose gradually increased to a therapeutic dose of 800 mg nightly	• severe reactive depression • mental state settles with quetiapine SR 800 mg nightly
**January 2023**	• ongoing diabetes, antihypertensive and thyroid treatment • quetiapine tapered and discontinued	• hyposomnia/insomnia, increased goal-directed behavior, impaired judgment, increased energy, reduced appetite • continued monitoring of mood/mental state/symptoms/signs of relapse
**February-June 2023**	• unmedicated bipolar disorder • the basal and total doses of insulin increased by 20% • antihypertensive therapy augmented by addition of ramipril 5 mg q.d. • ongoing thyroid treatment	• hypomanic-mixed-depressed episode • persisting significantly increased systolic and diastolic blood pressure values • follow-up HbA1c 7.26% (56 mmol/mol) in April 2023 • CGM time within target range 59%, time above 10.0 mmol/l 28%, time above 13.9 mmol/l 10% • average sensor glucose 9.3 mmol/l, glucose management indicator 7.3% (56 mmol/mol), glucose variability 37.4%
**July 2023**	• ongoing diabetes, antihypertensive and thyroid treatment • commencement of venlafaxine and quetiapine treatment • venlafaxine up titrated to 150 mg daily, discontinued after 23 days • quetiapine SR up titrated to 400 mg nightly • continued monotherapy with quetiapine SR 400 mg nightly • regular follow-up outpatient mental health appointments	• moderate depressive episodes • improvements in mood and glucose control with considerably reduced fluctuations

In January 2023, she underwent a common medical procedure that required a three-day inpatient hospital stay. The procedure went uncomplicated, and the patient was discharged and returned home and back to work. Two weeks later, she had an urgent review for not sleeping at all for several nights with extremely increased goal-directed behavior, impaired judgment, increased energy, and reduced appetite. It emerges that the patient had been reducing her quetiapine dose and completely discontinued her antipsychotic medication in mid-January 2023. Upon discussion, she revealed the reason for stopping her long−term psychopharmacological treatment. She admitted she wanted to make a new distracting problem to forget about her current another health issue she is extremely worried about. When asked to rate the helpfulness of her psychiatric medication prior to attempting discontinuation, she indicated that the medication had been extremely helpful. Since she refused any psychopharmacological alternatives to quetiapine for maintenance treatment of bipolar disorder, she agreed to continue monitoring symptoms, mood and mental state, and report any signs of relapse as soon as possible (**Table 2**).

**Figure 2:**
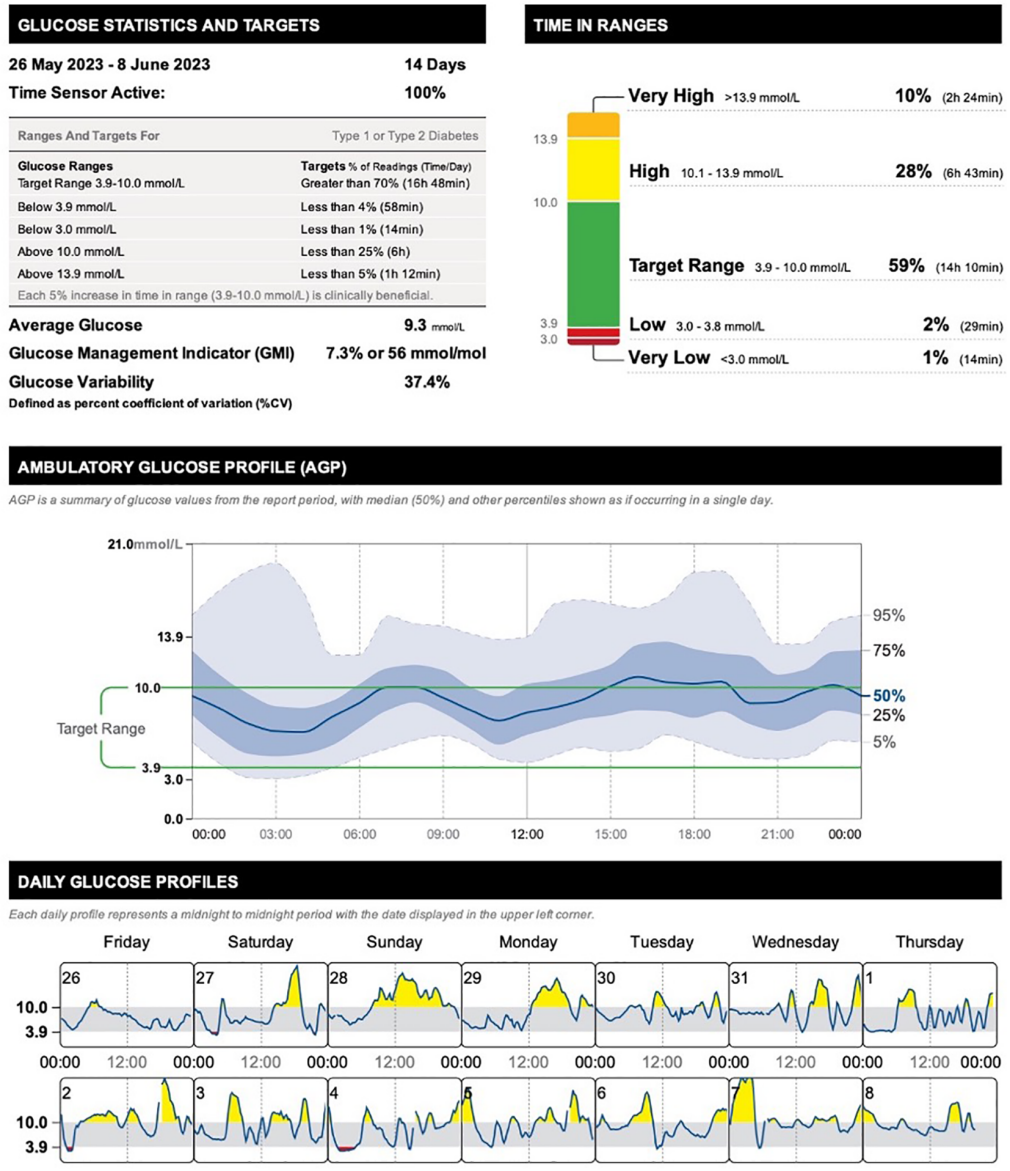
Summary of the continuous glucose monitoring results reflecting the period of unmedicated bipolar disorder (6-months following the discontinuation of quetiapine SR) in a 41-year-old female with comorbid type 1 diabetes mellitus.

Over the following six months of unmedicated bipolar disorder, the patient experiences a relatively unstable period alternating between hypomania lasting for several weeks to a less expansive, irritable mood, and a low mood. During the hypomanic episodes her sleep needs decreased to 1-4 hours per night, she was overtalkative, and her workmates expressed concerns that she had been overfamiliar with students. She reported feeling “fundamentally liberated” and accepted to be a little high. She did not report any delusional ideas and denied other psychotic phenomena. Over the coming weeks, her mood gradually normalized. Regarding the somatic comorbidities, following the discontinuation of quetiapine, the patient’s antihypertensive therapy was augmented by the addition of ramipril 5 mg q.d. because of the persisting significantly increased systolic and diastolic blood pressure values. The basal and total doses of insulin increased by 20%. Her follow-up labs were remarkable for significantly elevated NGSP HbA1c 7.26% (IFCC HbA1c 56 mmol/mol) in April 2023 (**Table 2**). **Figure 2** depicts the summary of the continuous glucose monitoring results reflecting the period of unmedicated bipolar disorder (6-months following the discontinuation of quetiapine). The glycemic control was considerably deteriorated, with parameters being outside the targets. The time within target range decreased to only 59%, time above 10.0 mmol/l increased to 28%, and time above 13.9 mmol/l increased to 10%. The average sensor glucose increased to 9.3 mmol/l, glucose management indicator deteriorated to 7.3% (56 mmol/mol), and glucose variability increased to 37.4%. The details of the ambulatory glucose profile and daily glucose profiles are depicted in **Figure 2** as well.

In June 2023, the patient’s mood worsened considerably, approaching moderate depressive episodes. In July 2023 (**Table 2**), the patient agreed to commence the psychopharmacotherapy with venlafaxine and quetiapine. Venlafaxine was gradually up titrated to 150 mg daily, and quetiapine SR was up titrated to 400 mg nightly to achieve its antidepressive, antimanic, stabilization, and prophylactic effects. Venlafaxine was discontinued after 23 days because of the onset of hypomanic symptoms at the end of July 2023. The patient continued monotherapy with quetiapine SR 400 mg nightly, and regular follow-up outpatient mental health appointments were scheduled. The follow-up appointments were spent on monitoring quetiapine dose and on reviewing side effects, goals, coping strategies, and progress. Support and psychoeducation were provided. The patient reported improvements in both mood and glucose control with considerably reduced fluctuations.

## Discussion

To our knowledge, this is a first report of the glycemic impact of medicated and unmedicated bipolar disorder on continuously monitored glucose levels in comorbid type 1 diabetes mellitus. Despite the known adverse metabolic effects of quetiapine, we observed more stable real-time continuous glucose values approaching normal glucose targets during the antipsychotic treatment with quetiapine compared to unmedicated stages of bipolar disorder that featured considerably higher glucose values and glucose variability consistent with the unstable mood.

In our previous report (4), we have demonstrated that targeting diabetes distress is critical to successful management of type 1 diabetes mellitus. Bipolar disorder as a psychiatric comorbidity represents and additional stressor through changes in mood that may precipitate dysglycemia in diabetes. The underlaying pathophysiology of mood-induced dysglycemia in diabetes remains less well understood. We have already reported the bidirectional links between bipolar disorder and type 2 diabetes mellitus (5). The information regarding associations between type 1 diabetes and bipolar disorder is scarce (6, 7). With respect to long-term glucose control, our case report shares a similarity with the previous case report by Chu and Liang (8) who reported the mood state as a blood glucose modulator in a patient with type 2 diabetes and bipolar disorder based on changes in HbA1c. Specifically, the authors report worsening of glucose control as indicated by increases in a three-month HbA1c that was related to bipolar depressive episodes, whereas improvements in HbA1c accompanied periods of hyperthymia and manic episodes (8). Associations between symptoms of depression and higher HbA1c levels have already been reported (9–11). A large Korean cohort study (12) has demonstrated that high glycemic variability and persistent hyperglycemia were associated with increased incidence of depression and anxiety disorders.

Novel glucose monitoring technologies have been developed to assist individuals with type 1 diabetes mellitus to make intensive therapeutic decisions 24/7, reduce risks, severity, and duration of hypo- and hyperglycemia, and thus help to achieve glucose targets with good precision and accuracy. With respect to the continuous glucose monitoring technology employed in the present case report, the FreeStyle Libre^®^ system utilizes artificial intelligence-based machine learning model to predict glucose variability and risk of imminent hypoglycemia in real-time. Our findings are in line with evidence from a systematic review (13) that has demonstrated a positive impact of the real-time continuous glucose monitoring on glycemic control.

This case report highlights the importance of the ongoing psychopharmacological treatment of bipolar disorder to prevent relapses of hypomania, mixed episodes, and depression, and thus help reduce the mood-induced reactivity, emotional urgency, and stress contributing to dysglycemia in type 1 diabetes mellitus. If not effectively treated, the “bipolar diabetes” is likely to progress to multiple psychiatric and somatic complications. Information about the course of daytime and nighttime continuous glucose values in type 1 diabetes mellitus comorbid with bipolar disorder has not been reported so far. The associated risks of dysglycemia (hyperglycemia along with the increased glucose variability) during various stages and phases of bipolar disorder and their treatment are not quantified at all. The novel findings of potential bidirectional links between the phases of bipolar disorder and the corresponding continuous glucose patterns reported here can enhance clinical decision-making and yield future innovative clinical research into “bipolar diabetes”.

Type 1 diabetes mellitus is a lifelong condition that requires an ongoing complex care addressing physical, mental, and emotional health. Specifically, it should target not only physical but also psychiatric comorbidities. Importantly, clinical management of bipolar disorder in persons with type 1 diabetes mellitus calls for balanced assessments of metabolic risks and glycemic benefits. Technology-enabled diabetes management solutions can facilitate this process. Given the risks of untreated bipolar disorder, discontinuation of psychopharmacotherapy should be discouraged. Advancing psychiatrists’ and diabetologists’ knowledge and skills may improve patient-clinician shared therapeutic decision making for persons with type 1 diabetes and comorbid bipolar disorder. Real-time continuous glucose monitoring can help reveal otherwise undetected glucose fluctuations related to mood changes, emotional urgency, and distress in various stages and phases of bipolar disorder. In addition, the continuous glucose profiles may serve as a motivational tool demonstrating a more stable glucose control in medicated bipolar disorder with a stable and normalized mood, and thus help increase an adherence to psychopharmacotherapy. It is likely that community mental health care may also help to better support patient choice and self-determination regarding the use and discontinuation of psychiatric medication, especially in case of crisis.

The patient has shared her perspective on the treatment she received and stopped receiving at some point to return back to it later: “I walked through hell after discontinuing my psychiatric medication, not sleeping at all, being crazy. My mood was unstable with extreme mood and blood sugar swings, with never-ending ups and downs. I was laughing and crying spells. Later, depressions with suicidal thoughts came back to me again…. There were times I beg for psychiatric treatment and its continuation despite my unwillingness. When I had decided to stop my medication, the only reason to do so was that I really needed to stop thinking about other health issues killing me at that time. I made my choice to artificially create a new problem that would distract me from the other problems. In addition, having a short-term experience to be a medication-free, I was worried that with a psychiatric medication, I would not be myself anymore. I would not be able to fully perceive my emotions. I would not be free anymore because of the decrease in my energy levels. I would stop flying high. I would have to fight with emotional barriers posed by medication, including my intimate life. I would have to bury or at least amputate my creativity. I would be a completely different person. After stopping my bipolar medication, I admitted the medication had been extremely helpful. With the medication, I found the stability that I needed in life. As a person living 25 years with type 1 diabetes, the experience of quetiapine discontinuation has taught me a lesson: My brain and mood have a joint control over my blood sugar that is beyond my control. So, it is my choice - it is up to me: To live longer and better with a stable, normalized mood and without significant diabetes complications or to have fluctuations in mood and fluctuations in blood sugar that will result in countless and serious problems”.

## Conclusion

The extent of dysglycemia during various stages and phases of bipolar disorder and its treatment remains unexplored in adults with type 1 diabetes mellitus. Continuous glucose monitoring allows to uncover hidden glucose patterns and associate them with mood changes, emotional urgency, and their treatment. Unmedicated bipolar disorder is characterized by less stable and elevated continuous glucose values compared to bimodal mood-stabilizing psychopharmacotherapy with quetiapine. Findings suggest that ongoing bipolar pharmacotherapy can alleviate mood-induced dysglycemia in type 1 diabetes mellitus. Continuous glucose profiles demonstrating a link between stability of glucose control and stability of mood can help boost the motivation of the patient and increase the adherence to psychopharmacotherapy. The enhanced patient-clinician shared clinical decision-making has a potential to yield future innovative clinical research into individually tailored treatment of “bipolar diabetes”. Optimal diabetes care is interdisciplinary and complex. It is to provide continuous medical care with multifactorial risk-reduction strategies to reach a goal of safe near-normal glucose levels and a good quality of life.

## Data availability statement

The raw data supporting the conclusions of this article will be made available by the authors, without undue reservation.

## Ethics statement

The studies involving humans were approved by the independent ethics committee of the Presov self-governing region. The studies were conducted in accordance with the local legislation and institutional requirements. The participants provided their written informed consent to participate in this study. Written informed consent was obtained from the individual(s) for the publication of any potentially identifiable images or data included in this article.

## Author contributions

DB: Conceptualization, Formal Analysis, Investigation, Methodology, Supervision, Writing – original draft, Writing – review & editing. MP: Conceptualization, Data curation, Formal Analysis, Investigation, Methodology, Project administration, Software, Writing – original draft, Writing – review & editing.

## References

[1] Grande I Berk M Birmaher B Vieta E. Bipolar disorder. Lancet (2016) 387(10027):1561–72. doi: 10.1016/S0140-6736(15)00241-X 26388529

[2] American, Psychiatric, Association. Diagnostic and Statistical Manual of Mental Disorders, Fifth Edition, Text Revision (DSM-5-TR). Washington, D.C.: American Psychiatric Association (APA) (2022).

[3] Huang YW Yin XS Li ZP. Association of the stress hyperglycemia ratio and clinical outcomes in patients with stroke: A systematic review and meta-analysis. Front Neurol (2022) 13:999536. doi: 10.3389/fneur.2022.999536 36119678 PMC9474893

[4] Pallayova M Taheri S. Targeting diabetes distress: the missing piece of the successful type 1 diabetes management puzzle. Diabetes Spectr (2014) 27(2):143–9. doi: 10.2337/diaspect.27.2.143 PMC4522882 26246771

[5] Breznoscakova D Pallayova M. Bipolar disorder and type 2 diabetes mellitus: A bidirectional relationship. Eur J Psychiatry (2022) 36(3):152–62. doi: 10.1016/j.ejpsy.2021.11.002

[6] Oulis P Karapoulios E Kouzoupis AV Masdrakis VG Kontoangelos KA Makrilakis K. Oxcarbazepine as monotherapy of acute mania in insufficiently controlled type-1 diabetes mellitus: a case-report. Ann Gen Psychiatry (2007) 6:25. doi: 10.1186/1744-859X-6-25 17922898 PMC2092422

[7] Sztein DM Lane WG. Examination of the comorbidity of mental illness and somatic conditions in hospitalized children in the United States using the kids’ Inpatient database, 2009. Hosp Pediatr (2016) 6(3):126–34. doi: 10.1542/hpeds.2015-0117 PMC5482715 26908823

[8] Chu CW Liang CS. Mood state as a blood glucose modulator in a patient with bipolar disorder and diabetes mellitus: A case report. Aust N Z J Psychiatry (2018) 52(10):1004–5. doi: 10.1177/0004867418787643 30014719

[9] Sajatovic M Gunzler D Einstadter D Thomas C McCormick RA Perzynski AT. Clinical characteristics of individuals with serious mental illness and type 2 diabetes. Psychiatr Serv (2015) 66(2):197–9. doi: 10.1176/appi.ps.201300538 PMC4315335 25642615

[10] Lustman PJ Anderson RJ Freedland KE de Groot M Carney RM Clouse RE. Depression and poor glycemic control: a meta-analytic review of the literature. Diabetes Care (2000) 23(7):934–42. doi: 10.2337/diacare.23.7.934 10895843

[11] Schmitt A McSharry J Speight J Holmes-Truscott E Hendrieckx C Skinner T. Symptoms of depression and anxiety in adults with type 1 diabetes: Associations with self-care behaviour, glycaemia and incident complications over four years - Results from diabetes MILES-Australia. J Affect Disord (2021) 282:803–11. doi: 10.1016/j.jad.2020.12.196 33601721

[12] Kwon M Lee M Kim EH Choi D-W Jung E Kim KY. Risk of depression and anxiety disorders according to long-term glycemic variability. J Affect Disord (2023) 343:50–8. doi: 10.1016/j.jad.2023.09.017 37734626

[13] Jaques-Albuquerque LT Dos Anjos-Martins E Torres-Nunes L Valério-Penha AG Coelho-Oliveira AC da Silva Sarandy VL. Effectiveness of using the freeStyle libre(^®^) system for monitoring blood glucose during the COVID-19 pandemic in diabetic individuals: systematic review. Diagn (Basel) (2023) 13(8):1499 doi: 10.3390/diagnostics13081499 PMC10137815 37189600

